# Sex-dependent dysregulation of human neutrophil responses by bisphenol A

**DOI:** 10.1186/s12940-020-00686-8

**Published:** 2021-01-07

**Authors:** Wioletta Ratajczak-Wrona, Marzena Garley, Malgorzata Rusak, Karolina Nowak, Jan Czerniecki, Katarzyna Wolosewicz, Milena Dabrowska, Slawomir Wolczynski, Piotr Radziwon, Ewa Jablonska

**Affiliations:** 1 Department of Immunology, Medical University of Bialystok, ul. Waszyngtona 15A, 15-269 Bialystok, Poland; 2 Department of Hematological Diagnostics, Medical University of Bialystok, ul. Waszyngtona 15A, 15-269 Bialystok, Poland; 3 Department of Biology and Pathology of Human Reproduction, Institute of Animal Reproduction and Food Research, Polish Academy of Sciences, Olsztyn, ul. M. Sklodowskiej-Curie 24a, 15-276 Białystok, Poland; 4 Department of Reproduction and Gynecological Endocrinology, Medical University of Bialystok, ul. M. Sklodowskiej-Curie 24a, 15-276 Bialystok, Poland; 5 Regional Centre for Transfusion Medicine, Bialystok, ul. M. Skłodowskiej - Curie 23, 15-950 Bialystok, Poland

**Keywords:** Neutrophils, Bisphenol A, 17β-estadiol, Nitric oxide

## Abstract

**Background:**

In the present study, we aimed to investigate selected functions of human neutrophils exposed to bisphenol A (BPA) under in vitro conditions. As BPA is classified among xenoestrogens, we compared its action and effects with those of 17β-estradiol (E2).

**Methods:**

Chemotaxis of neutrophils was examined using the Boyden chamber. Their phagocytosis and nicotinamide adenine dinucleotide phosphate hydrogen (NADPH) oxidase activity were assessed via Park’s method with latex beads and Park’s test with nitroblue tetrazolium. To assess the total concentration of nitric oxide (NO), the Griess reaction was utilized. Flow cytometry was used to assess the expression of cluster of differentiation (CD) antigens. The formation of neutrophil extracellular traps (NETs) was analyzed using a microscope (IN Cell Analyzer 2200 system). Expression of the investigated proteins was determined using Western blot.

**Results:**

The analysis of results obtained for both sexes demonstrated that after exposure to BPA, the chemotactic capacity of neutrophils was reduced. In the presence of BPA, the phagocytic activity was found to be elevated in the cells obtained from women and reduced in the cells from men. Following exposure to BPA, the percentage of neutrophils with CD14 and CD284 (TLR4) expression, as well as the percentage of cells forming NETs, was increased in the cells from both sexes. The stimulatory role of BPA and E2 in the activation of NADPH oxidase was observed only in female cells. On the other hand, no influence of E2 on the expression of CD14 and CD284, chemotaxis, phagocytosis, and the amount of NET-positive neutrophils was found for both sexes. The study further showed that BPA intensified NO production and iNOS expression in the cells of both sexes. In addition, intensified expression of all tested PI3K-Akt pathway proteins was observed in male neutrophils.

**Conclusions:**

The study demonstrated the influence of BPA on neutrophil functions associated with locomotion and pathogen elimination, which in turn may disturb the immune response of these cells in both women and men. Analysis of the obtained data showed that the effect of this xenoestrogen on the human neutrophils was more pronounced than E2.

## Background

The primary cell components of the innate immune system are phagocytes, which include neutrophils. They perform a wide spectrum of functions and thus constitute the first line of defense against pathogenic microorganisms and neoplastic cells. In addition, neutrophils serve as regulatory and effector cells in the mechanisms of the acquired immune response. The neutrophils present in the bloodstream are metabolically inactive and do not exhibit cytotoxic properties [1–6]. On receiving a chemical signal, the cells are stimulated and move to the site of infection/inflammation (chemotaxis). On their surface, neutrophils possess receptors that can recognize the molecular patterns of pathogens (PRMs) and can therefore identify foreign particles and differentiate them from their own. The PRMs include, inter alia, Toll-like receptors (e.g. TLR4), the ligand for which is lipopolysaccharide—LPS [1, 5, 7, 8]. One of the strategies used by neutrophils to kill pathogens is phagocytosis, which involves the stages such as “recognition” and “absorption” of the pathogen and formation of a phagolysosome. Inside the phagolysosome, the absorbed pathogen is killed by any of the two mechanisms: aerobic (associated with the production of reactive oxygen species (ROS) and reactive nitrogen species) and anaerobic (associated with the presence of proteins contained in the granules) [7, 9–13]. Another strategy used by neutrophils as the first line of defense to fight against pathogenic microorganisms is the creation of neutrophil extracellular traps—NETs. This phenomenon involves the release of the cellular nucleus contents (decondensed chromatin and histone proteins) together with the components of granularity (including myeloperoxidase (MPO)) and cell cytoplasm into the extracellular space. In addition to neutralizing and destroying the pathogens, NETs prevent their proliferation by creating a physical barrier [10, 14–20]. It is hypothesized that neutrophils prefer fast phagocytosis when they come in contact with small microorganisms, whereas when recognizing larger pathogens or aggregates of microorganisms, they activate the NET formation [21].

One of the reactive nitrogen radicals produced by the activated neutrophils is nitric oxide (NO). It is produced with the action of nitric oxide (NO) synthase enzymes, which include inducible nitric oxide synthase (iNOS), through the oxidation of L-arginine. The expression and activity of iNOS can be controlled by both endogenous and exogenous factors [22, 23]. Previous studies have demonstrated that bisphenol A (BPA) affects the production of NO by human neutrophils, with differences in the activity of NF-κB transcription factor in neutrophils depending on sex [24].

At present, the exposure of humans to BPA is increased, due to its wide application in the production of everyday products, such as food storage containers and fluids, plastic bottles, and infant feeding bottles. The human exposure to the compound occurs mainly via the oral route due to the fact that the BPA particles that were not polymerized completely during the production process are released from packaging to foods. BPA is also absorbed via the skin by contact with the thermal paper used to print receipts (e.g. ATM receipts) [25–28]. It has also been demonstrated to be present in many tissues and fluids including whole blood, umbilical cord blood, and urine [26, 29, 30]. An analysis conducted by the *National Health and Nutrition Examination Survey* showed the presence of BPA at a concentration of 1.8–660 nM in urine samples in the majority of US residents [31, 32].

BPA is a xenoestrogen—a chemical compound that resembles steroid hormones in its structure. It is classified among the endocrine-disrupting compounds (EDC) or endocrine disruptors. EDCs constitute a group of chemical compounds produced by people and do not occur naturally in the environment. These compounds modulate the intrasecretory activity by affecting the biosynthesis, metabolism, and hormone actions. The toxicity of these compounds cannot be accurately determined. The dosage and effect of EDCs seem to exhibit nonmonotonic relationships; both extremely low and high dosages can provide a maximum response, while the medium doses are associated with the concomitant absence of effect. This dosage–effect response of EDCs is often presented as a U-shaped curve, or also in the form of an inverted U-shaped curve, where medium dosing provides the maximum effect [33–38].

Data show that BPA may affect the signaling pathways associated with the nuclear (estrogen receptor (ER)-α and ERβ) and membrane receptors (GPR30) of estrogens [39]. Research studies indicate that the nuclear receptors are present in both female and male neutrophils [40].

In light of the current knowledge, it is assumed that human neutrophils are also the target cells of BPA. However, the molecular mechanisms of the action of this xenobiotic on neutrophils are not fully characterized. Earlier works have shown the effect of BPA on neutrophil immunophenotypes as well as its gender-dependent effects on the intracellular killing of neutrophils associated with the release of serine proteases [41, 42]. Therefore, we conducted further studies in women and men to find answers to the following questions: Can BPA affect the basic neutrophil functions? If so, does this effect depend on gender? To better understand the basis of the molecular action mechanism of this xenobiotic on the functions of the studied leukocyte population, especially those related to NO synthesis and release, we assessed the role of another intracellular signaling pathway, PI3K-Akt, which is crucial for the activation of NF-κB transcription factor signaling pathway. As the PI3K-Akt pathway is activated by phospho-PI3K, phospho-Akt (T308), and phospho-Akt (S473) proteins, we presumed that the evaluation of their expression in the cytoplasmic fraction of BPA-exposed neutrophils can confirm or deny the participation of this signaling pathway in iNOS activation.

## Methods

### Reagents

BPA, superoxide dismutase from bovine erythrocytes, cytochrome C from equine heart, Griess reagent, nitroblue tetrazolium (NBT), N-formyl-Met-Leu-Phe (fMLP), and latex were purchased from Merck Millipore (Burlington, MA). May–Grünwald and Giemsa stains were obtained from Aqua-med. Polimorphoprep™ (Axis Shield) and phosphate-buffered saline (PBS) with or without CaCl_2_ and MgCl_2_ ions were supplied by Thermo Fisher Scientific (Waltham, MA). Wortmannin was purchased from Calbiochem (San Diego, CA). ICI 182.780 (ICI/Fulvestrant, 98% pure) and 17β-estradiol (E2) were obtained from Sigma-Aldrich.

### Materials

The study involved a group of 15 healthy voluntary blood donors [5 women (in the follicular phase of the menstrual cycle) aged 22–25 years and 10 men aged 23–30 years], who were recruited from the Regional Centre for Transfusion Medicine, Bialystok, Poland. All the donors were healthy, and did not smoke or drink alcohol in the last 48 h before blood collection. The donors also had no history of chronic diseases or immunological deficiencies in the last 5 years. All the donors gave written informed consent before blood donation. For the study, 9 ml of whole blood was taken from a vein in the arm of each donor and transferred to a test tube with anticoagulant (NH Sodium Heparin anticoagulant, Greiner Bio-One GmbH). The human material study was carried out according to the recommendations of the Bioethics Committee of the Medical University of Białystok (Resolution No. R-I-002/396/2019, R-I-002/333/2017). All the experiments were performed in accordance with Good Laboratory Practice.

E2 was used at a physiological concentration of 100 pM. In the earlier work [41], we found that the mean BPA concentration in the serum samples of healthy people was, respectively, 14.94 nM in women and 17.17 nM in men. Based on this observation, BPA was used at two concentrations in the present study: 16 nM—mean BPA concentration in donor blood; 1.6 μM—a 100-fold higher BPA concentration than the mean concentration in the human serum, which referred to high exposure to this xenoestrogen. Data from the literature suggest that BPA is accumulated in humans from different products (Table 1).

**Table 1:** Literature data concerning the sources of exposure of humans to BPA [43]

Source of exposure	Route of BPA penetration to the organism	The most vulnerable group	Estimated daily dose [ng/day]
Nutrition	Digestive tract	Adults	1560–10,453
Thermal paper	Skin	Cashiers	1303–40,590

### Neutrophil isolation

The preliminary cell isolation—separation of polymorphonuclear cells (PMNs) (contains 91% of neutrophils) from the peripheral blood mononuclear cells (contains 94% of lymphocytes)—was carried out by centrifuging the blood samples in density gradient using Polymorphprep™ reagent (AXIS-SHIELD PoC AS, Oslo, Norway). Briefly, the blood was carefully layered on the reagent, the amount of which was equal to the amount of blood used for separation. Subsequently, the sample was centrifuged at 400 g for 30 min at room temperature. Cells were counted in the Bürker chamber after staining the nuclei with Türk’s solution. To obtain a pure neutrophil fraction (99.9%) from the PMN fraction, an additional isolation step was carried out using magnetic separation (MACS® Separator) with antibodies and magnetic CD16 MicroBeads (catalog no. 130–045-701, Miltenyi Biotec) [42]. The survival of neutrophils was evaluated using trypan blue under a light microscope, which was found to be 97%.

### Assessment of cell capacity of chemotaxis by Boyden chamber

The chemotactic ability of neutrophils was assessed using the Boyden chamber (Neuro Probe, Gaithersburg, MD), a chemoattractant solution (fMLP), and a filter with a pore size of 5 μm diameter. The Boyden chamber consists of two compartments separated by a filter. One of the compartments was filled with fMLP—neutrophils will be attracted to the direction of increasing concentration of chemoattractant. The second compartment was filled with 50,000 neutrophils suspended in PBS with CaCl_2_ and MgCl_2_ ions. The isolated cells were previously preincubated for 30 min in an incubator with 5% CO_2_ (Nuarie™ US Autoflow, Plymouth, MN) at 37 °C without any additional substances, or with BPA (16 nM or 1.6 μM) or E2 (100 pM). After both compartments were filled, the chamber was incubated for an hour in an incubator with 5% CO_2_ (Nuarie™ US Autoflow) at 37 °C. Then, the solutions were removed, and the filter was placed on a slide. The filter was stained using the May–Grünwald–Giemsa staining method and evaluated under a light microscope with oil immersion. All the neutrophils found on the filter and those sticking to the filter pores were counted. The percentage of cells capable of chemotaxis was calculated from the proportion of 50,000 cells as 100%.

### Assessment of cell capacity of phagocytosis by Park’s method with latex beads

Blood collected on heparin was centrifuged at 2000 rpm for 5 min. The neutrophils collected from the “leukocyte coat” fraction were first incubated in an incubator at 37 °C, with a constant flow of 5% CO_2_ (Nuarie™ US Autoflow), for 30 min without or with BPA (16 nM or 1.6 μM) or E2 (100 pM). Latex (exogenous beads) was added to the suspension, which was absorbed into the cytoplasm of cells that were capable of phagocytosis. Then, the suspension was incubated in an incubator with 5% flow of CO_2_ (Nuarie™ US Autoflow) at 37° temperature for 30 min and in at room temperature for the next 30 min. After incubation, smears were prepared, fixed, and stained using the May–Grünwald–Giemsa staining method. The smears were evaluated under oil immersion in a light microscope, and the neutrophils were differentiated into four classes based on the amount of the absorbed latex beads: class 0—neutrophils without absorbed latex beads, class I—neutrophils containing 1–10 latex beads, class II—neutrophils containing 11–30 latex beads, and class III—neutrophils containing more than 30 latex beads in the cytoplasm. This allowed the quantitative (percentage of cells capable of phagocytosis) and qualitative (Social Cohesion and Reconciliation (SCORE) index)) assessment of phagocytosis. The SCORE index was obtained by multiplying the group’s name and the number of neutrophils in the group.

### Assessment of nicotinamide adenine dinucleotide phosphate hydrogen (NADPH) oxidase activity by Park’s test with NBT

The use of Park’s test with NBT enables analyzing the NADPH oxidase activity of human neutrophils. The method is based on the reduction of NBT absorbed into the cell cytoplasm into insoluble blue formazan crystals.

Whole-blood samples were incubated for 30 min without or with BPA (16 nM or 1.6 μM) or E2 (100 pM). Then, NBT was added, moreover, latex beads which stimulated cells to absorption were added into NBT stimulated samples. The samples were then incubated for 15 min at 37 °C with a constant flow of 5% CO_2_ (Nuarie™ US Autoflow) and 15 min at room temperature. Then, smears were prepared on slides, fixed, and stained using the May–Grünwald–Giemsa staining method. The smears were evaluated under oil immersion in a light microscope. The results were expressed as the percentage of cells with formazan in the spontaneous test or as the percentage of cells with formazan and latex in the stimulated test.

### Neutrophil extracellular traps

Isolated neutrophils were suspended in a culture medium containing Rosewell Park Memorial Institute 1640, antibiotics (100 U penicillin/ml and 50 ng streptomycin/ml), and serum (4%). They were stimulated to form NETs on 96-well culture plates. For this purpose, the cells were introduced in an amount of 5 × 10^4^ per well. Then, they were incubated for 60 min at 37 °C under 5% CO_2_ (Nuarie™ US Autoflow) in the absence or presence of LPS (10 μM), BPA (16 nM or 1.6 μM), or E2 (100 pM). Hoechst 3342 dye (Invitrogen) (at a concentration of 1 μg/ml prepared in PBS), which allows the detection of DNA, and antihuman MPO antibody (Life Technologies), which allows detecting the main NET component, were added to the samples. The analysis of NET formation in the wells was carried out under a fluorescence microscope (IN Cell Analyzer 2200, GE Healthcare Life Sciences) using the IN Cell Analyzer Workstation program. The stained cells were counted in nine 20× microscopic fields per section. The results were expressed as the percentage of cells showing the NET formation related to all the neutrophils of an image. For statistical analysis, the mean value of nine images was used for calculating the average values for each sample.

### Cluster of differentiation

Isolated neutrophils were suspended in Hank’s Balanced Salt Solution (HBSS) 1X culture medium (5 × 10^5^ cells/ml) in the presence of serum (4%) and antibiotics (100 U penicillin/ml and 50 ng streptomycin/ml). The microplate (Microtest III-Falcon) was incubated for 2 h at 37 °C, with a constant flow of 5% CO_2_ (Nuarie™ US Autoflow), in the presence of LPS (10 μM), BPA (16 nM or 1.6 μM), or E2 (100 pM). Then, the plate was centrifuged, and after washing and suspending in PBS, the neutrophils were assessed for the expression of CD14 and CD284.

The expression of cluster of differentiation (CD) antigens on the cells was determined by the direct fluorescence method using Canto II flow cytometer (Becton Dickinson) and monoclonal antibodies (Becton Dickinson). Briefly, 20 μl/5 μl of anti-CD14/anti-CD284 monoclonal antibodies, respectively, was added to 50 μl of the neutrophils suspended in PBS. After 30 min of incubation in the dark, the cells were resuspended in PBS and analyzed for 30 min using the FASCSDiva software. Simultaneously, an isotypic sample was prepared which was used for analyzing the obtained results.

### Assessment of total NO concentration in neutrophil supernatants by Griess reaction

Isolated neutrophils were suspended in HBSS 1X culture medium (5 × 10^5^ cells/ml) in the presence of serum (4%) and antibiotics (100 U penicillin/ml and 50 ng streptomycin/ml). The microplate (Microtest III-Falcon) was incubated for 2 h at 37 °C, with a constant flow of 5% CO_2_ (Nuarie™ US Autoflow), without or with BPA (16 nM or 1.6 μM) or E2 (100 pM). Some cells were previously preincubated for 60 min with a wortmannin inhibitor (0.1 μM, Calbiochem, San Diego, CA) or with ICI 182.780 (1 μM, ICI/Fulvestrant, Sigma-Aldrich, 98% pure). Then, the plate was centrifuged and the culture supernatants were used to assess the total NO concentration. The cell precipitate was collected and used for assessing protein expression by Western blot.

NO production by neutrophils was assessed using an indirect method in which the concentration of NO_2_^−^ ions in culture supernatants was measured according to the Griess reaction. The total NO concentration was calculated as the sum of the nitrate (III) and nitrate (V) concentrations. The Griess reaction was used for detecting only nitrates (III) and involved two stages. In the first stage, the neutrophil supernatants were incubated for 30 min with cadmium to reduce nitrates (V) to nitrates (III). In the second stage, the Griess reagent was added, and the absorbance was read at 540 nm using a spectrophotometer. The concentration of NO products was expressed in μM (5 × 10^5^ cells in 270 ml of supernatant).

### Western blot

The cytoplasmic fraction of neutrophils was isolated using the NucBuster™ Protein Extraction Kit (Merck). The isolated cytoplasmic protein was suspended in 2X Laemmli Sample Buffer (Bio-Rad) with βME (Bio-Rad). Then, the protein was applied to polyacrylamide gel in an amount of 10 μg/well, and electrophoresis under denaturing conditions (SDS-PAGE) was performed in Mini-PROTEAN® Tetra Cell (Bio-Rad). Using the Transfer Buffer solution, the separated proteins were electrophoretically transferred from the gel to a nitrocellulose membrane on a 0.45 m roll (Bio-Rad) in the Mini-PROTEAN® Tetra Cell apparatus. The next stages of the procedure were carried out in the SNAP i.d.® 2.0 (Millipore) apparatus. The nitrocellulose membrane was blocked in 1X Tris-buffered saline (TBS) with 1% Casein Blocker (Bio-Rad) and incubated for 10 min at room temperature with primary antibodies: anti-p-PI3K goat polyclonal antibody, anti-p-Akt1/2/3 (B-5) mouse monoclonal antibody, anti-p-Akt1/2/3 (Ser473) rabbit polyclonal antibody, and anti-iNOS rabbit polyclonal antibody (1:100, Santa Cruz Biotechnology). The membrane was rinsed three times in TBS (Bio-Rad) with Tween® 20 (Sigma). Then, it was incubated for 10 min at room temperature with appropriate secondary antibodies labeled with alkaline phosphatase (1:5000, Santa Cruz Biotechnology) and rinsed three times again. Immunoreactive strips were obtained by adding BCIP®/NBT Liquid Substrate System (Sigma). The intensity of the stained strips was evaluated using the ImageJ software (NIMH, Bethesda, MD). The results were expressed in arbitrary units.

### Statistical analysis

STATISTICA version 13.3 program (StatSoft, Inc., Tulsa, OK) was used for statistical analysis. Data are presented as mean ± standard deviation (SD). Levene test was chosen to check the homogeneity of variations. The normal distribution of data was tested by the Shapiro–Wilk test. For the comparison between groups, Student’s *t*-test, analysis of variance (ANOVA) with Tukey post hoc test, Mann-Whitney test or the Kruskal-Wallis analysis of variance (ANOVA) were applied for parametric and non-parametric data, respectively. A significance level of *p* < 0.05 was considered statistically significant.

## Results

**Fig. 1:**
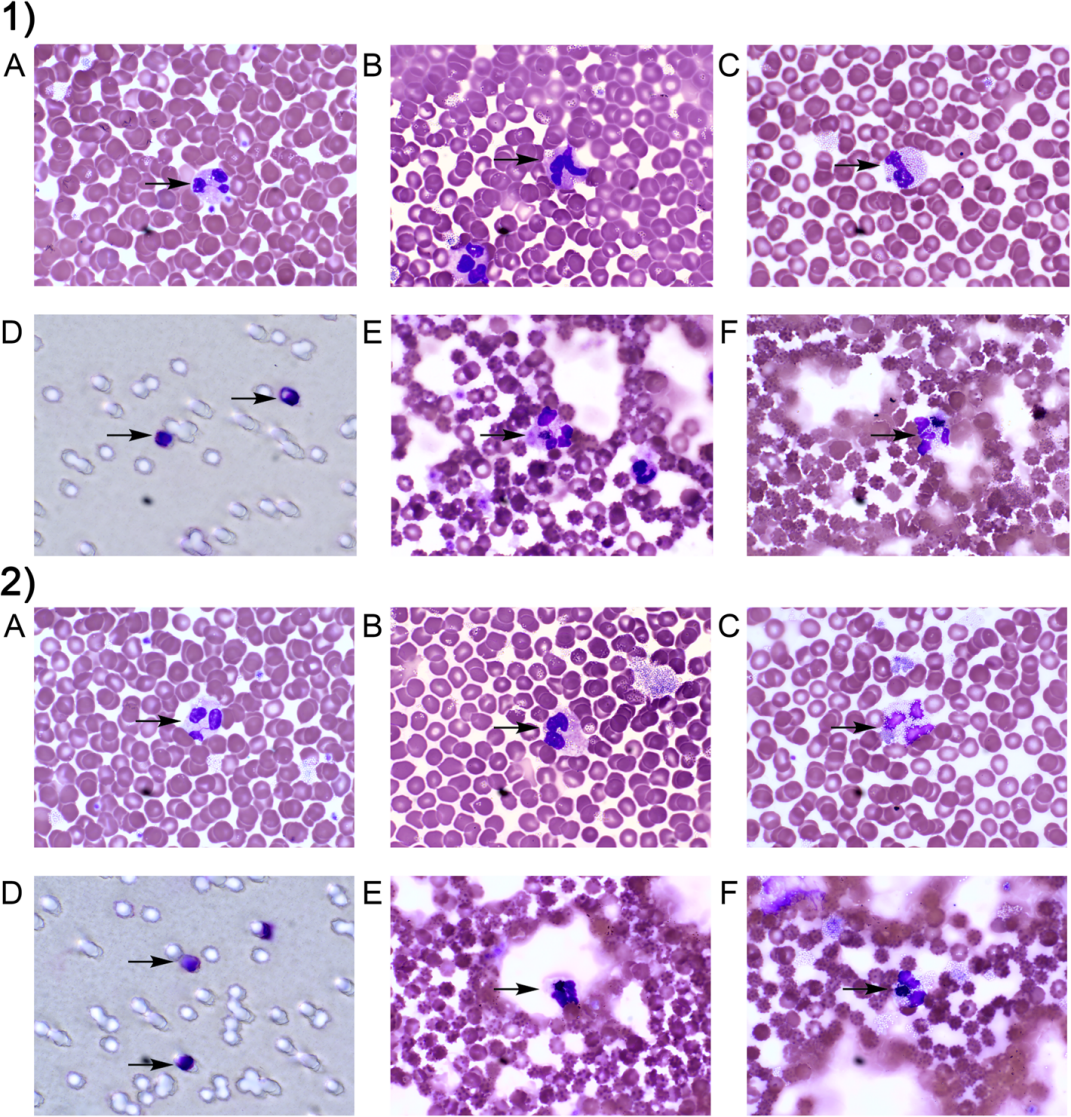
Representative photographs of neutrophils (incubated without (1) or with BPA at a concentration of 16 nM (2)). Slides were stained using May–Grünwald and Giemsa stains. Neutrophils were counted manually in a light microscope. Magnification × 100. **a**–**c** Neutrophil ability of phagocytosis was assessed using Park’s test with latex beads. **a** Neutrophil in the first group with up to 10 latex beads in the cytoplasm. **b** Neutrophil in the second group with 11–30 latex beads in the cytoplasm. **c** Neutrophil in the third group with more than 30 latex beads in the cytoplasm. **d** Neutrophil ability of chemotaxis was evaluated using the Boyden chamber. Arrow indicates the cell that passed through the membrane pore, while arrow with star indicates the cell staying in the membrane pore. **e** and **f** Activity of NADPH oxidase in neutrophils was evaluated using the NBT test. **e** Neutrophil with formazan crystal in the spontaneous test. **f** Neutrophil with latex beads and formazan crystal in the cytoplasm in the stimulated test

**Fig. 2:**
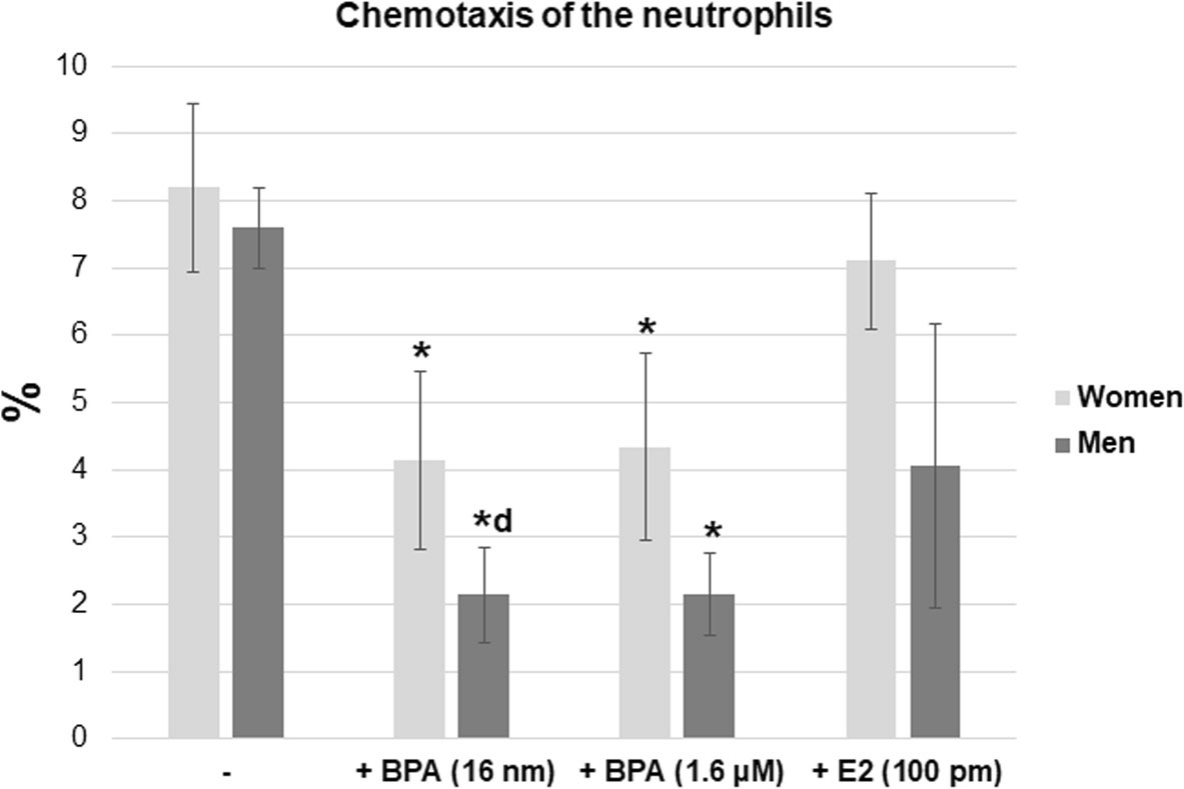
Chemotaxis of the neutrophils of women and men. Value significantly different between: *cells without and with BPA (*p* < 0.05); d women and men (*p* < 0.05)

After the exposure to BPA (16 nM and 1.6 μM), a reduction in the percentage of neutrophils capable of chemotaxis was observed in samples from both women and men (Fig. 1d, Fig. 2). No differences were noted in the percentage of neutrophils capable of chemotaxis in the presence of E2 (100 pM) in both sexes as compared to the cells incubated without the compound. In addition, no statistically significant differences were seen between female and male cells with respect to the chemotactic ability, which is one of the main functions of neutrophils (Fig. 1d, Fig. 2).

**Fig. 3:**
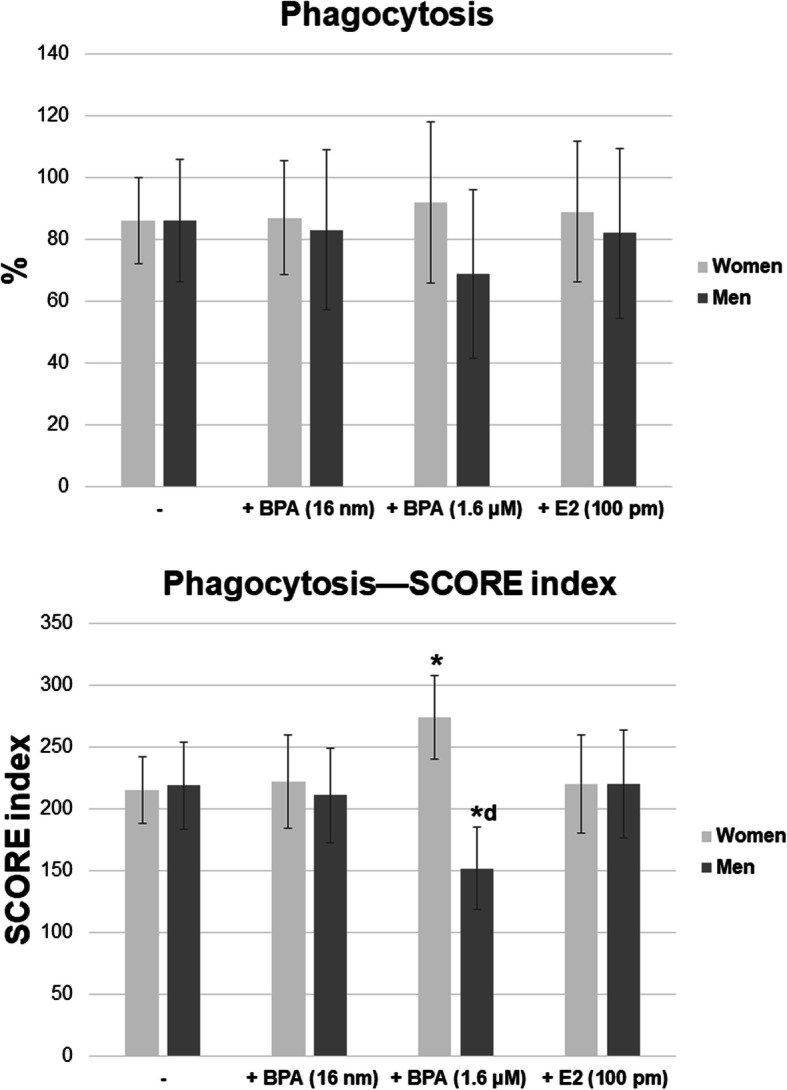
Phagocytosis–the percentage of phagocytic cells in women and men. Phagocytosis–SCORE index in women and men. Value significantly different between: *cells without and with BPA (*p* < 0.05); d women and men (*p* < 0.05)

Exposure of female and male neutrophils to BPA at all tested concentrations and E2 (100 pM) did not lead to any change in the percentage of cells with phagocytic ability (Fig. 1a-c, Fig. 3).

In the case of female cells, it was observed that exposure to 1.6 μM BPA caused an increase in the phagocytic activity, as shown by the SCORE index, in comparison with the unexposed cells (Fig. 3). In contrast to the results obtained for female neutrophils, the incubation of male neutrophils with 1.6 μM BPA resulted in a decrease in their phagocytic activity, as shown by the SCORE index (Fig. 1a-c, Fig. 3).

On the other hand, 16 nM BPA and E2 (100 pM) had no effect on the phagocytosis of the examined male and female cells (Fig. 1a-c, Fig. 3).

Analysis of the results of both sexes showed that the phagocytic activity of male cells incubated in the presence of BPA (1.6 μM) was reduced, as indicated by the SCORE index, compared to female neutrophils (Fig. 1a-c, Fig. 3).

**Fig. 4:**
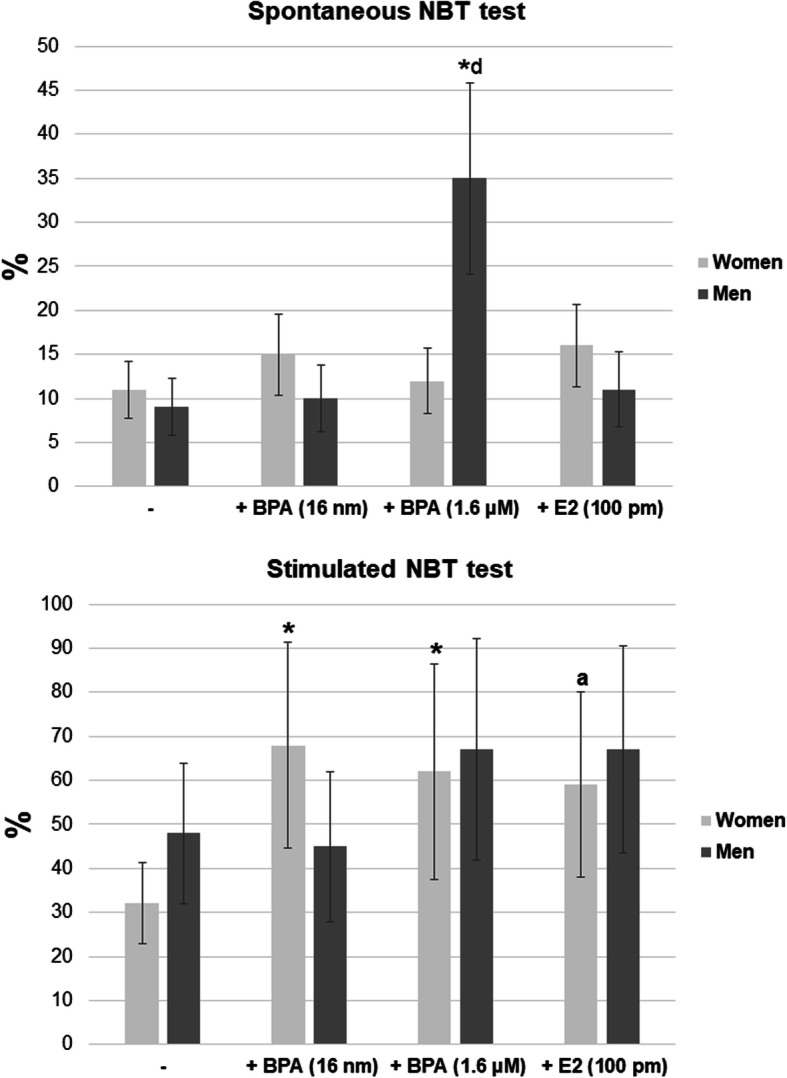
Spontaneous NBT test in women and men. Stimulated NBT test in women and men. Value significantly different between: *cells without and with BPA (*p* < 0.05); a cells without and with E2 (*p* < 0.05); d women and men (*p* < 0.05)

After incubation with BPA (16 nM and 1.6 μM) and E2 (100 pM), the proportion of NBT-positive cells was not increased in the spontaneous NBT test in the case of female neutrophils as compared to the cells incubated without this compound. On the other hand, in the case of male neutrophils, the percentage of NBT-positive cells was significantly higher when the cells were incubated in the presence of BPA at 1.6 μM, compared to both female neutrophils and cells incubated without the compound (Fig. 1e-f, Fig. 4).

The stimulated test showed an increased proportion of NBT-positive cells for female neutrophils incubated in the presence of BPA at both concentrations as well as E2, compared to the cells incubated without the compound. By contrast, no changes in the percentage of NBT-positive cells in the presence of BPA (16 nM and 1.6 μM) or E2 (100 pM) were observed for male neutrophils (Fig. 1e-f, Fig. 4).

No statistically significant differences in the percentage of NBT-positive cells were found between female and male neutrophils (Fig. 1e-f, Fig. 4).

**Fig. 5:**
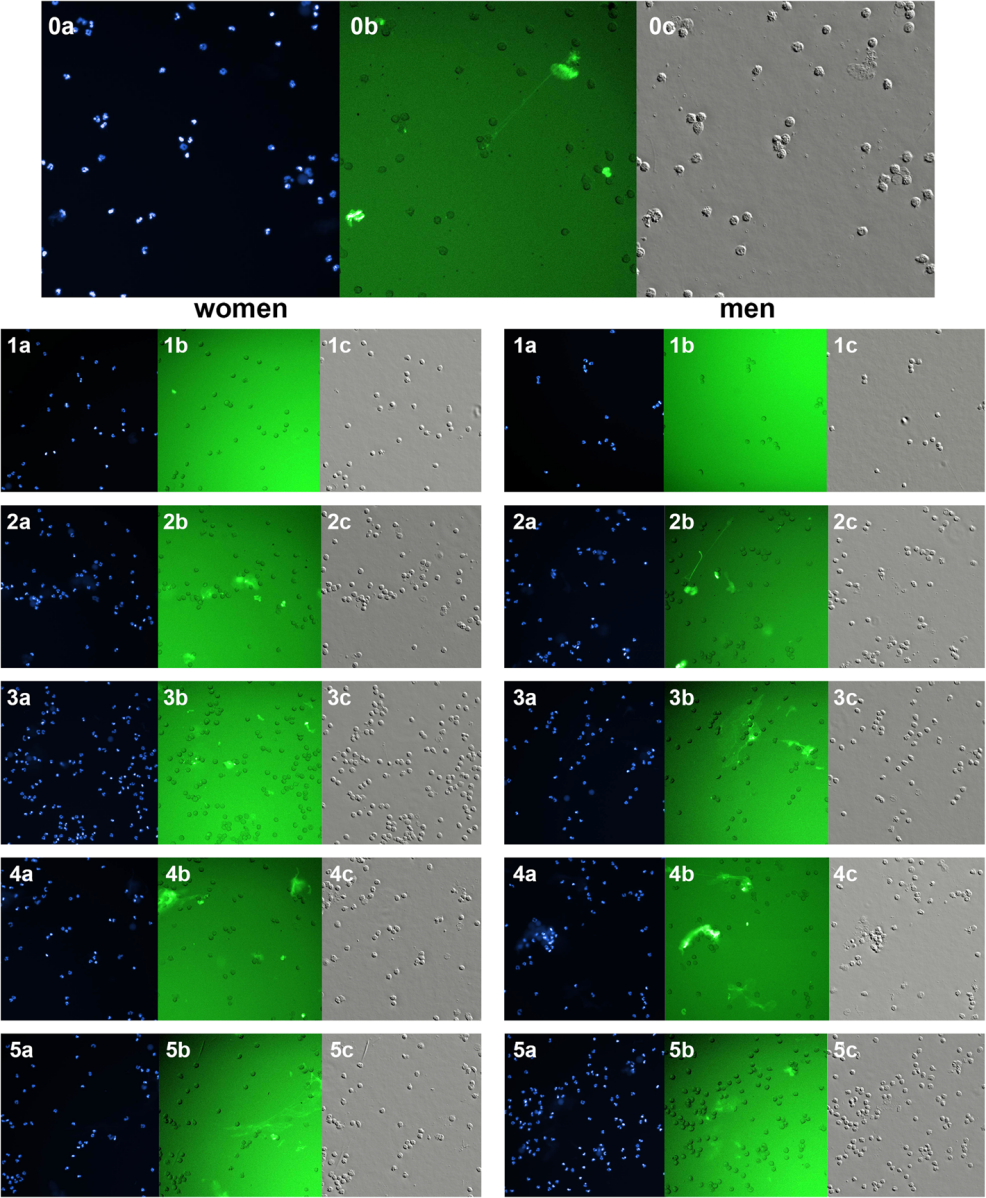
Representative images of NETs induced in vitro by LPS, BPA, or E2 in human neutrophils. Neutrophils from healthy donors (women and men) were stimulated with the indicated reagents and analyzed by a fluorescence microscope and In Cell Analyzer Workstation (c–differential interference contrast setting). Cells were stained with Hoechst 3342 against chromatin (a–*blue*) and anti-MPO antibody (b–*green*) to verify the NET production. Original magnification × 20. Representative images of neutrophils are shown. 1–unstimulated neutrophils; 2–LPS (10 μg/ml) stimulation; 3–BPA (16 nM) stimulation; 4–BPA (1.6 μM) stimulation; 5–E2 (100 pM) stimulation

**Fig. 6:**
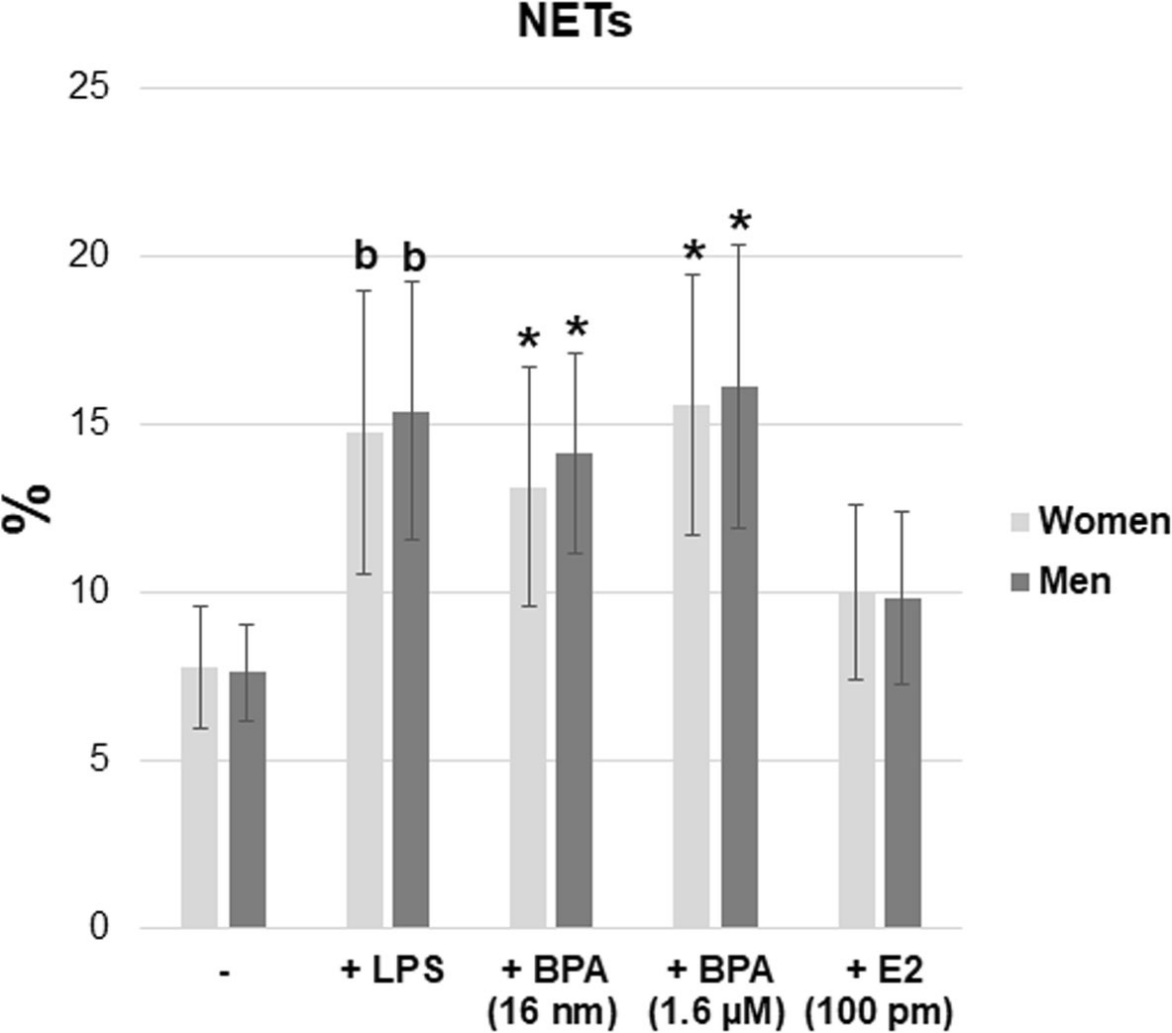
Summary of the average values of NET-forming neutrophils. Value significantly different between: *cells without and with BPA (*p* < 0.05); b–cells incubated only with LPS and cells incubated without BPA and LPS (*p* < 0.05)

Stimulation of female and male neutrophils with LPS caused an increase in the number of NETs compared to the networks generated by the nonstimulated cells. In contrast to E2 (100 pM), an increased percentage of NET-forming cells was observed in the presence of BPA (16 nM and 1.6 μM) for both female and male neutrophils as compared to the untreated cells (Fig. 5, Fig. 6).

**Fig. 7:**
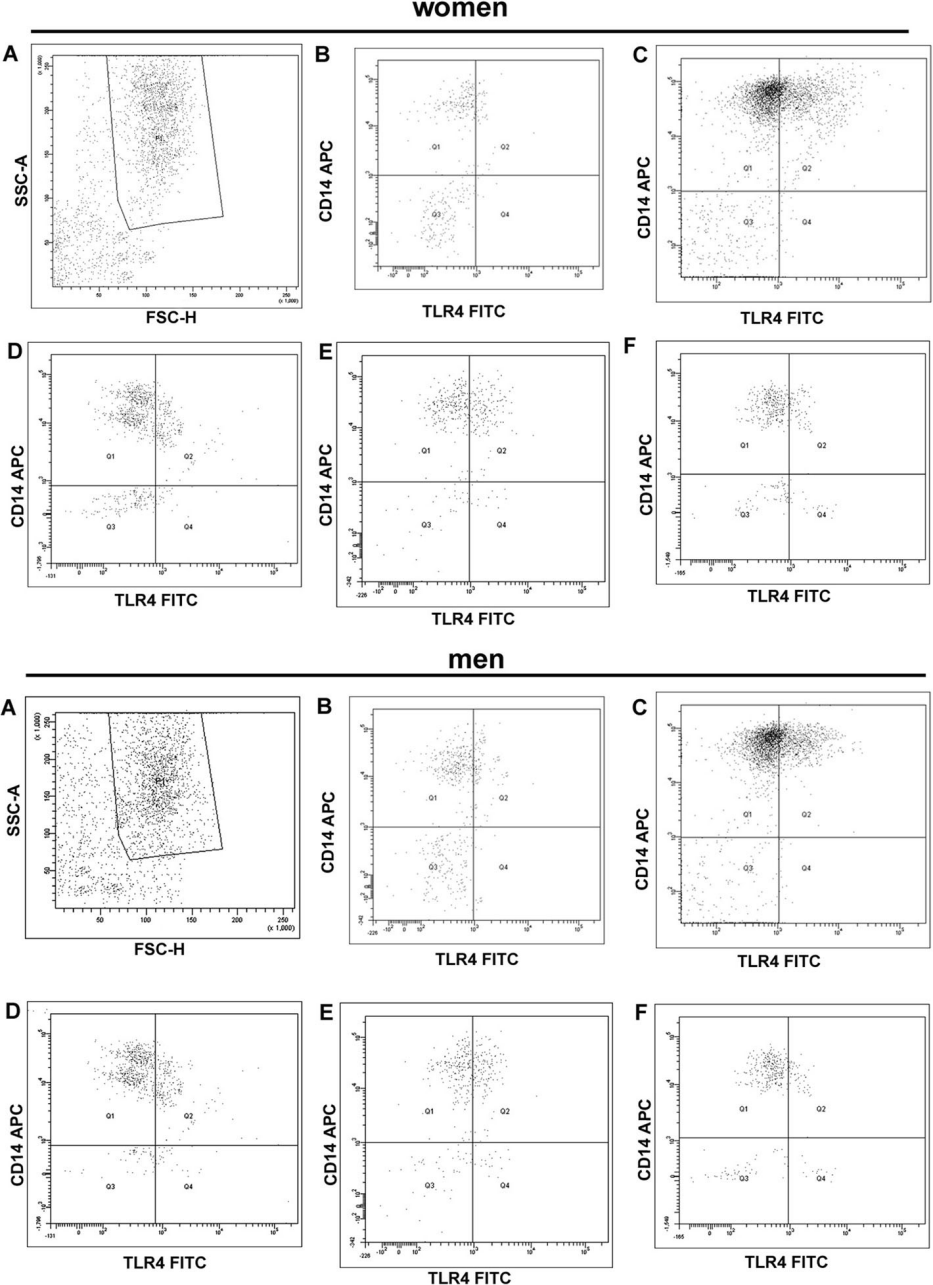
Representative flow cytometry plots demonstrating the expression of CD antigens (CD14 and CD284 (TLR4)) on neutrophils: **a** neutrophils (SSC/FSC); **b** neutrophils without BPA and LPS; **c** cells incubated only with LPS; **d** cells incubated only with BPA (16 nM); **e** cells incubated only with BPA (1.6 μM); **f** cells incubated only with E2 (100 pM)

The cell membrane of neutrophils isolated from both men and women displayed the expression of TLR4, CD284, and CD14 (Fig. 7).

A higher percentage of neutrophils had TLR4 in both male and female samples after BPA (16 nM and 1.6 μM) application in comparison to the cells incubated without this xenoestrogen. No changes were seen in the percentage of neutrophils expressing this molecule in the presence of E2 (100 pM) (Table 2).

**Table 2:** Alterations in CD284 (TLR4) and CD14 in human neutrophils. Neutrophils were treated for 2 h without or with BPA (16 nM or 1.6 μM) or LPS (10 μg/ml)

	CD284 (TLR4)				CD14			
	% of positive cells		MFI		% of positive cells		MFI	
	Women mean ± SD [%]	Men mean ± SD [%]	Women mean ± SD	Men mean ± SD	Women mean ± SD [%]	Men mean ± SD [%]	Women mean ± SD	Men mean ± SD
**PMN**	6.27 ± 2.03	6.14 ± 1.99	4.26 ± 3.22	3.87 ± 4.02	52.15 ± 13.2	54.6 ± 14.58	65.64 ± 19.29	68.01 ± 7.98
**PMN + LPS**	32.8^b^ ± 8.41	28.3^b^ ± 8.09	25.93 ± 4.88	24.75^b^ ± 5.74	84.7^b^ ± 17.12	92.8^b^ ± 18.07	121.54 ± 25.22	135.22 ± 32.22
**PMN + BPA** **(16 nM)**	16.3^a^ ± 4.29	15.33^a^ ± 4.99	9.43 ± 3.33	8.48 ± 2.22	78.1^a^ ± 16.03	79.2^a^ ± 16.65	91.23 ± 10.11	93.82 ± 21.91
**PMN + BPA** **(1.6 μM)**	24.75^a^ ± 7.99	22.7^a^ ± 6.13	18.77 ± 6.55	15.43 ± 8.77	59.6 ± 18.01	55.1 ± 15.64	72.22 ± 24.22	69.11 ± 13.22
**PMN + E2** **(100 pM)**	7.95 ± 1.97	6.2 ± 1.78	5.11 ± 4.21	3.99 ± 0.98	58.12 ± 19.22	60.6 ± 21.3	71.22 ± 32.22	74.38 ± 17.77

Value significantly different between: ^a^cells without and with BPA (*p* < 0.05), ^b^cells incubated only with LPS and cells incubated without BPA and LPS (*p* < 0.05). *MFI* mean fluorescence intensity

Exposure of female and male neutrophils to BPA (16 nM and 1.6 μM) led to an increase in the percentage of cells with CD14 expression as compared to the untreated cells. In the presence of E2 (100 pM), no changes were observed in the percentage of neutrophils with CD14 expression (Table 2).

LPS stimulation of both male and female neutrophils resulted in an increase in the percentage of cells with TLR4 and CD14 expression, compared to cells not stimulated by LPS (Table 2).

**Fig. 8:**
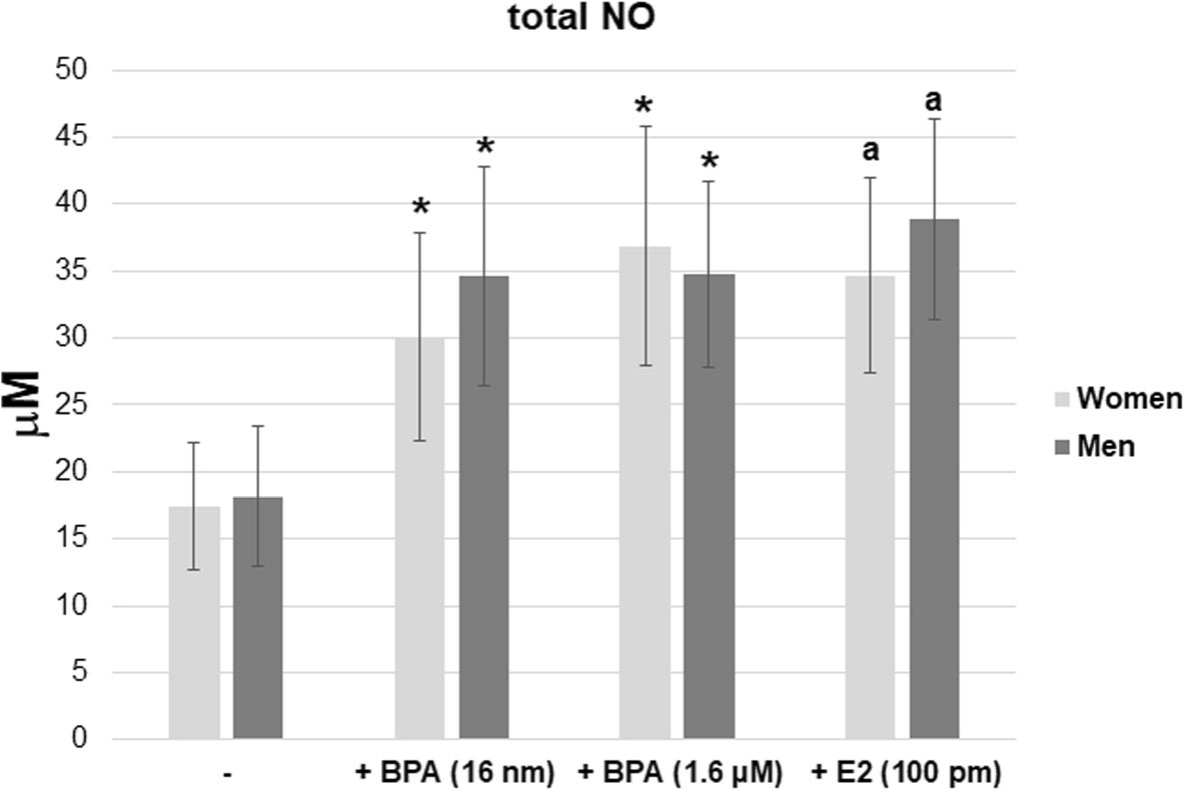
Concentrations of total NO in the neutrophils supernatants. Neutrophils were treated without or with BPA (16 nM or 1.6 μM) or with E2 (100 pM) for 2 h, and then the supernatants were subjected to nitrite assay. Value significantly different between: *cells without and with BPA (*p* < 0.05); a–cells without and with E2 (*p* < 0.05)

The female and male neutrophils showed an increase in the release of NO in the presence of BPA (16 nM and 1.6 μM) and E2 (100 pM), compared to the neutrophils incubated without the compound. No statistically significant differences were found in total NO concentrations between the male and female neutrophil supernatants (Fig. 8).

However, in the presence of BPA (16 nM and 1.6 μM) and E2 (100 pM), higher concentrations of total NO were observed in male neutrophil supernatants compared to the supernatants of the cells incubated without the compound (Fig. 8).

**Fig. 9:**
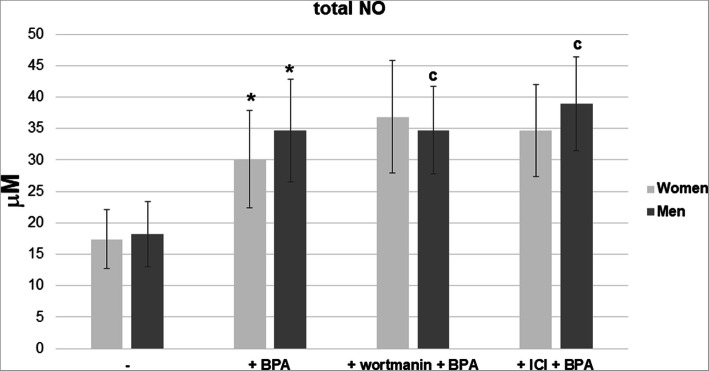
Concentrations of total NO in the neutrophils supernatants. Neutrophils were treated without or with BPA (16 nM) or coincubated with wortmannin (0.1 μM) or ICI 180.720 (1 μM) for 2 h, and then the supernatants were subjected to nitrite assay. Value significantly different between: *—cells without and with BPA (*p* < 0.05); c–BPA-treated cells preincubated with or without inhibitor (*p* < 0.05)

In order to determine the participation of PI3K in iNOS production by neutrophils exposed to BPA, its concentration in the presence of a selective inhibitor was evaluated. Furthermore, experiments with the ER inhibitor (ICI) were performed to understand the role of ERs in this process (Fig. 9).

In the presence of wortmannin or ICI inhibitor, no changes in total NO concentration were observed in female neutrophil supernatants compared to the supernatants of the cells incubated without the inhibitor. In turn, lower concentrations of this radical were found in the male neutrophil supernatants compared to the cells incubated without the inhibitor (Fig. 9).

**Fig. 10:**
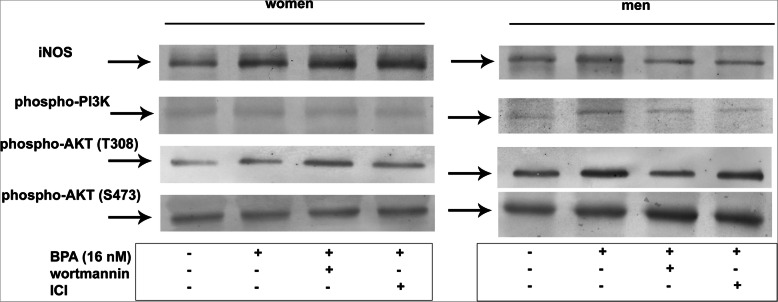
Expression of iNOS, phospho-PI3K, phospho-Akt (T308), and phospho-Akt (S473) in human neutrophils. Neutrophils were treated with or without wortmannin (0.1 mM) or ICI 180.720 (1 μM) for 1 h before the addition of BPA (16 nM). The cytoplasmic fractions obtained from the cells were used to detect the protein levels of iNOS, phospho-PI3K, phospho-Akt (T308), and phospho-Akt (S473) by Western blot. These results are representative of five independent experiments

The incubation of female and male neutrophils with 16 nM BPA caused higher expression of iNOS and phospho-Akt (T308) in the cytoplasmic fraction as compared to the cells incubated without the xenoestrogen. In contrast to female neutrophils, male neutrophils exposed to BPA showed an increased expression of phospho-PI3K and phospho-Akt (S473) (Fig. 10, Table 3).

**Table 3:** Band intensity was quantified using ImageJ software and expressed in arbitrary units (A.U)

Arbitrary units (A.U).								
	Women				Men			
	PMN	PMN + BPA (16 nM)	PMN + wormanin + BPA (16 nM)	PMN + ICI + BPA (16 nM)	PMN	PMN + BPA (16 nM)	PMN + wormanin + BPA (16 nM)	PMN + ICI + BPA (16 nM)
	mean ± SD	mean ± SD	mean ± SD	mean ± SD	mean ± SD	mean ± SD	mean ± SD	mean ± SD
**iNOS**	73,602 ± 16,664.48	121391^a^ ± 19,436.22	173411^c^ ± 17,211.74	195392^c^ ± 18,359.31	79,979 ± 15,221.77	138417^a^± 18,654.85	99982^c,d^ ± 18,963.14	101273^c,d^ ± 15,469.23
**phospho-PI3K**	74,673 ± 18,580.1	83,177 ± 17,469.22	82,881 ± 165,487.53	71,545 ± 17,365.74	91,245 ± 18,362.84	143493^a^ ± 22,145.66	99005^c^ ± 178,965.21	91727^c^ ± 21,518.1
**phospho-Akt (T308)**	165,281 ± 36,346.2	215469^a^ ± 48,176.63	365813^c^ ± 67,795.12	273775^c^ ± 58,443.75	350054^d^ ± 63,511.34	437066^a,d^ ± 8615.52	370979^c^ ± 56,128.76	361734^c^ ± 69,581.48
**phospho-Akt (S473)**	227,547 ± 50,060.34	237,535 ± 39,882.35	300272^c^ ± 41,046.24	299886^c^ ± 52,976.06	369437^d^ ± 70,581.77	418813^a,d^ ± 8786.34	534478^c,d^ ± 72,441.78	496401^c,d^ ± 89,208.22

Data shown are mean and standard deviation (SD) of five independent experiments. Value significantly different between: ^a^cells without and with BPA (*p* < 0.05) or ^c^BPA -treated pre-incubated with or without inhibitor (*p* < 0.05)

^d^women and men (*p* < 0.05)

To confirm the participation of PI3K in the induction of iNOS expression in neutrophils exposed to BPA, experiments with a selective inhibitor of this kinase (wortmannin) were carried out.

In the presence of the PI3K inhibitor, a higher expression of iNOS and phospho-Akt (T308) in the cytoplasmic fraction was found in female neutrophils, whereas a lower expression of the same was found in male neutrophils compared to the cells incubated without the inhibitor. In contrast to female neutrophils, the exposure of male neutrophils to BPA led to a decrease in phospho-PI3K expression compared to the cells incubated without the inhibitor. Both female and male neutrophils showed an increased phospho-Akt (S473) expression (Fig. 10, Table 3).

Experiments with the ER inhibitor (ICI) were also carried out to examine the role of ERs in the induction of the tested proteins.

In the presence of the ICI inhibitor, a higher expression of iNOS in the cytoplasmic fraction was found in the female neutrophils exposed to BPA, whereas a lower expression of iNOS was observed in male neutrophils. In the case of phospho-PI3K, no changes were noted in the expression in the cytoplasmic fraction of female neutrophils. In contrast to female neutrophils, after the application of ICI inhibitor, the male neutrophils exposed to BPA showed a lower expression of phosphor-PI3K in the cytoplasmic fraction compared to the cells incubated without an inhibitor. No changes in phospho-Akt (T308) expression were seen in both female and male neutrophils. However, a higher expression of phospho-Akt (S473) was observed in the cells of both sexes compared to the cells incubated without the inhibitor (Fig. 10, Table 3).

## Discussion

The results of the present study demonstrated that the effect of BPA modulates the basic functions of human neutrophils.

This study, which was conducted at our laboratory, showed that based on sex, BPA exerted different effects on the chemotaxis of neutrophils as compared with estradiol. The inhibitory effect of BPA on the chemotaxis of these cells was also reported by Balistrieri’s team and the studies carried out on neutrophils isolated from people chronically exposed to dichlorodiphenyltrichloroethane—an insecticide classified as EDC. It was found that EDC compounds, including BPA, reduce the ability of neutrophils to carry out chemotaxis, phagocytosis, and aerobic killing. The same group of researchers also observed that the impairment of neutrophil functions in these individuals was associated with an increased incidence of diseases such as upper respiratory tract infections [44, 45].

The results of our study analyzing the influence of estradiol (applied at the physiological concentration) on the phagocytic activity of neutrophils are consistent with those obtained by other scientists. Shibuya’s team did not observe any effect of estradiol on the zymosan-induced phagocytic activity of neutrophils. However, the researchers showed that high concentrations of estradiol inhibited MPO degranulation from cytoplasmic neutrophil granules, while the physiological concentrations of estrogen reinforced this process [46].

The mechanism of action of EDCs in cells is quite complex. BPA may function via a few intermediates including nuclear receptors (e.g. ERs, androgen receptor, PPAR) and act as their antagonists or agonists. Depending on the concentration of EDCs and their binding affinity to receptors, exposure to these compounds may lead to different effects. Moreover, one of the modulators of EDCs action is endogenous hormones, which are natural ligands for nuclear receptors [38, 47–49]. Their concentrations vary between sexes, and in women, they change during each phase of the menstrual cycle. Thus, we observed differences between women and men in the direction of action of BPA and estradiol related to the phagocytic activity of the exposed neutrophils. The inhibitory effect of BPA (1.6 μM) on the phagocytic activity of neutrophils as measured by the SCORE index is consistent with the results presented by Balistrieri et al. [44]. These researchers demonstrated the inhibitory effect of BPA on the neutrophils’ ability to phagocytose and kill *Staphylococcus aureus*. However, a different trend was found for neutrophils in women.

Liao et al. [50] suggested that the mechanism of BPA action on neutrophil activity may result from the direct influence of this xenoestrogen on TLR, including TRL4, or indirect influence affecting cell signaling. Our study demonstrated that BPA activates TLR4 in neutrophils. Therefore, it can be assumed that one of the mechanisms of BPA action on these cells is the direct action, which triggers an inappropriate neutrophil-associated immune response in the case of defense against pathogens.

In the light of the earlier observation that the action of BPA increased the percentage of neutrophils expressing CD14 antigen, the fact that the interactions of this xenoestrogen with CD14, as well as with TLR4, may consequently lead to the elevated expression of proinflammatory genes and cytokines seems to be of paramount importance [50, 51].

It is important to note that, in this study, we demonstrated the regulatory stimulating role of BPA (at doses of 16 nM and 1.6 μM) and estradiol in the activation of NADPH oxidase measured by the NBT stimulated test in female neutrophils. On the other hand, while evaluating the NADPH oxidase activity in the NBT spontaneous test, we observed the stimulating effect of BPA (at 1.6 μM) only in male neutrophils. These results indicating the stimulating effect of BPA on the generation of superoxide anion radicals can be explained by the possibility that BPA can influence the messenger RNA (mRNA) expression of NADPH oxidase subunits: p47phox and p67phox. This has been shown by Watanabe et al. [52] who demonstrated the effect of BPA (at concentrations of 2–12 μM) on the mRNA expression of these subunits.

Therefore, the obtained results suggest that the differences in the phagocytic activity and activation of NADPH oxidase between female and male neutrophils under the influence of BPA may result from a stronger affinity of this compound to ERβ than ERα. Molero et al. [40] showed that estradiol upregulated both ERα and ERβ in female neutrophils but only ERα in male cells. BPA is considered to be a relatively weak xenoestrogen as compared to E2, and its affinity to ERα and ERβ is 1000–10,000 times smaller. However, it shows a 10 times higher affinity to ERβ than ERα [53]. Interestingly, estrogens might also have a significant impact on the sex-dependent effects of BPA on neutrophil functions. This hormone increases the number of peripheral blood neutrophils, but simultaneously suppresses its proinflammatory action. Because of the various levels of estrogens in women and men, there will be differences in their neutrophil functions. Moreover, the different basal concentrations of estrogens in individuals’ bloodstream may modulate the primary level of BPA sensitivity [54, 55].

In this study, we also investigated whether BPA may exhibit other effects on the neutrophils of healthy people, depending on its concentration. The results showed that only at the concentration of 1.6 μM, BPA modulated neutrophil functions related to absorption. These observations appear to be particularly concerning in the case of people who are professionally exposed to BPA, considering the fact that the phagocytic activity is the first line of defense against many pathogens [9–13].

Efficient production of superoxide anion radicals is crucial for the oxygen-killing mechanism involving the production of ROS, which has a direct cytotoxic effect on pathogens. Moreover, the activation of NADPH oxidase is an important element regulating the NETosis process, which refers to the generation of NETs that neutralize the pathogens immobilized in them [56, 57]. In the present study, we could not observe the effect of estradiol on the counts of NET-forming cells at the physiological concentration used (100 pM). However, different results were reported by Yasuda et al., who determined that E2 at a high concentration (5 μM, observed during pregnancy) intensified the NETs formation [58]. Furthermore, contrasting results to ours were obtained by some authors with 1 and 3 h longer incubation of neutrophils with E2. On the other hand, the present study revealed the influence of BPA on NETs formation by neutrophils in both sexes. Considering the disadvantages or the ill effects of the traps, excessive generation of networks in people exposed to this xenoestrogen may lead to the development of inflammatory diseases, including autoimmune disorders [59, 60]. Additionally, previous studies indicated that BPA modulates the epigenetic DNA modification by influencing DNA methylation and the posttranslational modification of histones. EDCs have been reported to cause intergenerational hereditary susceptibility to DNA-related disorders. It has also been suggested that maternal exposure to EDCs may contribute to fetal changes that will have genetic consequences in future generations [61].

Available data show that E2 leads to NO generation via a mechanism associated with ERs [62]. Our earlier study showed that the action of BPA leads to an increased expression of ERs in human neutrophils. Experiments using BPA also showed the same results, indicating its stimulating effect, as observed with estradiol, on the generation of NO by neutrophils in both sexes. Therefore, it can be concluded that the effect of estradiol observed in such a short period of time (after 2 h), as well as that of BPA, may result from a nongenomic mechanism [63–65]. This leads to the question: Does the generation of NO with iNOS participation result from the activity of the PI3K-Akt pathway in these cells? Interestingly, in this study, with BPA at the concentration measured in the blood (16 nM), we observed the involvement of the PI3K-AKT pathway in this process only for male cells. In the case of female neutrophils, we found BPA involvement in the threonine 308 phosphorylation of Akt kinase. These data show that the phosphorylation of threonine and serine residues of Akt kinase may occur not only independent of the participation of kinases but also autocatalytically [66]. Moreover, based on the changes in NO production and iNOS expression in the presence of wortmannin inhibitor (PI3K pathway inhibitor) in male and female neutrophils exposed to BPA, it can be assumed that NO synthesis with iNOS involvement in these cells is associated with PI3K. However, depending on the sex, it plays a different role in this process.

More importantly, the results obtained in the presence of ICI suggest that BPA affects NO regulation in female and male cells by acting on ERs. However, the sex-dependent differences observed in the effects of BPA on iNOS regulation, which is largely related to ERα and ERβ, seem to result from the activation of separate control pathways by BPA in both receptors.

## Conclusions

To sum up, the results of our study indicate a significant influence of BPA on the basic functions of neutrophils, in both women and men, which may affect the first phase of immune response associated with defensive reactions against pathogens and cancer cells (Table 4). Furthermore, neutrophils isolated from women were found to be more susceptible to the effect of BPA in terms of oxygen-dependent killing, compared to those obtained from men. The variable results of the tests conducted on human neutrophils exposed to BPA indicated the stronger effect of this xenoestrogen compared to E2. Moreover, the differences in those results between women and men indicated sex-dependent differences in the mechanisms of the xenoestrogen’s influence on neutrophils.

**Table 4:** Summary of the effect of BPA or E2 on neutrophil functions

Function	Test	Tested substance					
		BPA (16 nM)		BPA (1.6 μM)		E2 (100 pM)	
		Women	Men	Women	Men	Women	Men
Chemotaxis	Boyden chamber	↓	↓	↓	↓	↔	↔
Phagocytosis	Park’s method with latex (%)	↔	↔	↔	↔	↔	↔
Phagocytosis	Park’s method with latex (SCORE)	↔	↔	↑	↓	↔	↔
NADPH oxidase activity	Park’s test with NBT/NBT spontaneous	↔	↔	↔	↑	↔	↔
NADPH oxidase activity	Park’s test with NBT/NBT stimulated	↑	↔	↑	↔	↑	↔
NETs	Fluorescence microscopy	↑	↑	↑	↑	↔	↔
CD284 (TLR4)	Flow cytometry	↑	↑	↑	↑	↔	↔
CD14	Flow cytometry	↑	↑	↔	↔	↔	↔
NO	Griess reaction	↑	↑	↑	↑	↑	↑

Legend: ↑—increase, ↓—decrease, ↔—no statistical change. *Abbreviations*: *BPA* bisphenol A, *E2* 17β-estradiol, *NETs* neutrophil extracellular traps, *NO* nitric oxide, *NBT* nitroblue tetrazolium, *SCORE* Social Cohesion and Reconciliation

Considering the results presented above, one of the characteristic features of BPA (EDCs) seems to be its nonmonotonic dose–effect relationship, which makes it difficult to determine the specific direction of its effects on neutrophils, and in particular, the mechanism that could probably lead to these effects. The possibility of simultaneous interaction of multiple xenoestrogen compounds should also be taken into account, as it may result in the overlapping of effects, their intensification, or mutual inhibition. Therefore, further research is necessary to clarify the changes that may occur under the influence of BPA and the resulting effects on human health. Moreover, in order to reduce exposure to BPA, it appears that we should handle plastic packaging properly, including the avoidance of heavy-duty cleaners as well as rough scrubbers that can damage the surface of the packaging.

## Data Availability

The datasets used and/or analyzed during the current study are available from the corresponding author on reasonable request.

## Abbreviations

BPA: Bisphenol A
EDC: Endocrine-disrupting compounds
E2: 17β-estradiol
NO: Nitric oxide
iNOS: inducible nitric oxide synthase
NETs: Neutrophil extracellular traps
CD: Cluster of differentiation
ER: Estrogen receptor
